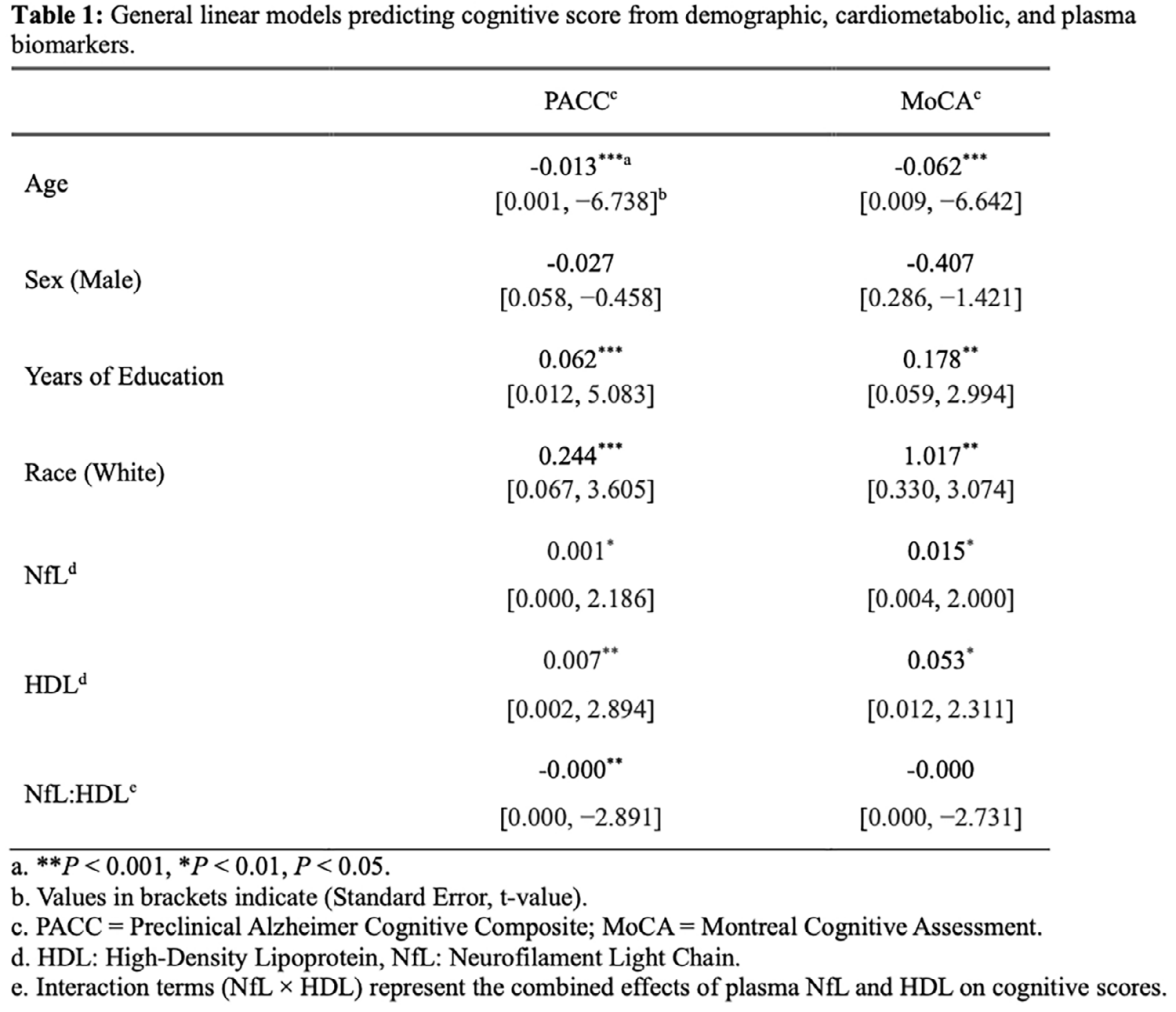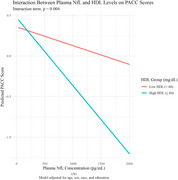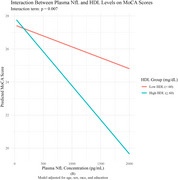# Plasma NfL and Cognitive Functioning in Older Adults: The Moderating Role of HDL Cholesterol

**DOI:** 10.1002/alz70861_108232

**Published:** 2025-12-23

**Authors:** Ramkrishna Kumar Singh, Semere Bekena, Yiqi Zhu, Carlos Cruchaga, Steven E Arnold, Beau Ances, Ganesh M. Babulal

**Affiliations:** ^1^ Washington University School of Medicine, Saint Louis, MO USA; ^2^ NeuroGenomics and Informatics Center, Washington University School of Medicine, St. Louis, MO USA; ^3^ Hope Center for Neurological Disorders, Washington University in St. Louis, St. Louis, MO USA; ^4^ The Charles F. and Joanne Knight Alzheimer Disease Research Center, St Louis, MO USA; ^5^ Washington University School of Medicine, St. Louis, MO USA; ^6^ Massachusetts General Hospital, Harvard Medical School, Boston, MA USA; ^7^ Institute of Public Health, Washington University School of Medicine, Saint Louis, MO USA; ^8^ University of Johannesburg, Johannesburg, Gauteng Province South Africa

## Abstract

**Background:**

Plasma neurofilament light chain (NfL), a biomarker of neuroaxonal damage, is associated with cognitive decline and neurodegeneration. High‐density lipoprotein (HDL) cholesterol, known for its anti‐inflammatory and antioxidative properties, has been linked to cognitive performance in aging populations. However, the relationship between HDL and neurodegeneration remains unclear. This study examines how HDL influences the relationship between plasma NfL and cognitive performance across mid to late adulthood, using data from individuals aged 36 years and older.

**Method:**

This cross‐sectional study analyzed baseline data from 418 participants of the Aging Adult Brain Connectome (AABC) study (mean age=66.5 years, 55.4% female). Plasma NfL was measured using the Simoa platform, and HDL cholesterol via enzymatic colorimetric assays. Cognitive performance was assessed with the Montreal Cognitive Assessment (MoCA) and Preclinical Alzheimer’s Cognitive Composite (PACC). Generalized linear models assessed the interaction between NfL and HDL on cognitive outcomes, adjusting for age, sex, race, and education. Sensitivity analyses included APOE ε4 genotype, body mass index, total cholesterol, LDL, and triglycerides.

**Result:**

The interaction between NfL and HDL was statistically significant across both cognitive outcomes (Table 1). Higher HDL levels consistently moderate the negative association between NfL and cognitive performance. For individuals with elevated HDL (≥60 mg/dL), increasing NfL concentrations were associated with lower scores on MoCA (β = ‐0.00019, *p* =0.006) and PACC (β = ‐0.00004, *p* =0.004). These associations were visualized in interaction plots (Figure A & B), which illustrate stronger inverse relationships between NfL and cognitive scores at higher HDL levels. The interaction effects remained robust after adjusting for additional covariates, including APOE ε4 genotype, BMI, total cholesterol, LDL, and triglycerides.

**Conclusion:**

HDL cholesterol moderates the association between plasma NfL and cognitive performance in older adults. Elevated HDL levels enhance the negative impact of NfL on cognition, challenging the assumption that HDL is uniformly protective in aging. As a modifiable factor, HDL offers a clinically actionable target in efforts to delay cognitive decline. These findings align with the United States Department of Health and Human Services (HHS) priorities on biomarker‐based strategies and underscore the value of integrating metabolic and neurodegenerative biomarkers for improved cognitive risk profiling and targeted interventions.